# Monitoring gestational weight gain and prepregnancy BMI using the 2009 IOM guidelines in the global population: a systematic review and meta-analysis

**DOI:** 10.1186/s12884-020-03335-7

**Published:** 2020-10-27

**Authors:** Jose Alberto Martínez-Hortelano, Iván Cavero-Redondo, Celia Álvarez-Bueno, Miriam Garrido-Miguel, Alba Soriano-Cano, Vicente Martínez-Vizcaíno

**Affiliations:** 1 Universidad de Castilla-La Mancha, Social and Health Care Research Center, Santa Teresa Jornet s/n, 16071 Cuenca, Spain; 2 Universidad Politécnica y Artística del Paraguay, Asunción, Paraguay; 3 Faculty of Health Sciences, Universidad Autónoma de Chile, Talca, Chile

**Keywords:** Gestational weight gain, Institute of Medicine gestational weight gain guidelines, Prepregnancy body mass index, Trend

## Abstract

**Background:**

Previous studies have reported a high prevalence of excessive gestational weight gain (GWG) in women with prepregnancy BMI classified as overweight and obese. However, the joint evidence regarding GWG and prepregnancy BMI in the worldwide population has not been synthesized. Thus, this systematic review and meta-analysis aimed to estimate global and regional mean GWG and the prevalence of GWG above, within and below 2009 Institute of Medicine (IOM) guidelines. Second, we aimed to estimate global and regional prepregnancy BMI and the prevalence of BMI categories according to World Health Organization (WHO) classification.

**Methods:**

We searched Medline, Embase, the Cochrane Library and Web of Science to identify observational studies until 9 May 2018. We included studies published from 2009 that used 2009 IOM guidelines, reporting data from women in general population with singleton pregnancies. The 2009 IOM categories for GWG and the WHO categories for prepregnancy BMI were used. DerSimonian and Laird random effects methods were used to estimate the pooled and their respective 95% confidence intervals (95% CIs) of the mean and by category rates of GWG and prepregnancy BMI, calculated by global and regions.

**Results:**

Sixty-three published studies from 29 countries with a total sample size of 1,416,915 women were included. The global prevalence of GWG above and below the 2009 IOM guidelines, was 27.8% (95% CI; 26.5, 29.1) and 39.4% (95% CI; 37.1, 41.7), respectively. Furthermore, meta-regression analyses showed that the mean GWG and the prevalence of GWG above guidelines have increased. The global prevalence of overweight and obesity, was 23.0% (95% CI; 22.3, 23.7) and 16.3% (95% CI; 15.4, 17.4), respectively. The highest mean GWG and prepregnancy BMI were in North America and the lowest were in Asia.

**Conclusions:**

Considering the high prevalence of GWG above the 2009 IOM guidelines and women with overweight/obesity and their continuously increasing trend in most regions, clinicians should recommend lifestyle interventions to improve women’s weight during reproductive age. Due to regional variability, these interventions should be adapted to each cultural context.

**Trial registration:**

Prospectively registered with PROSPERO (CRD42018093562).

**Supplementary information:**

**Supplementary information** accompanies this paper at 10.1186/s12884-020-03335-7.

## Background

In 2009, the Institute of Medicine (IOM) updated the international gestational weight gain (GWG) cut-off points published in 1990 [1] based on the prepregnancy body mass index (BMI) following the BMI classification of the World Health Organization (WHO) [2, 3]. The recommended amount of GWG in the 2009 IOM guidelines was 12.5-18 kg, 11.5–16 kg, 7–11.5 kg, and 5–9 kg for women with prepregnancy BMI classified as underweight (< 18.5 kg/m^2^), normal weight (18.5–24.9 kg/m^2^); overweight (25–29.9 kg/m^2^) and obese (≥ 30 kg/m^2^) respectively. The main change in the IOM cut-off points included an updated limitation of the recommended amount of GWG to improve pregnancy outcomes in women with obesity and the change in the classification criteria of prepregnancy BMI according to the WHO classification [3, 4]. This fact modified the GWG categoy prevalence and the advice about GWG that public health practitioners gave to women.

Gestational weight gain above IOM guidelines and prepregnancy overweight/obesity have been continuously increasing during recent decades although data differ across countries [5, 6]. Recent studies have reported high rates of GWG above the IOM guidelines and the prepregnancy overweight/obesity in Europe over 36 and 29%, respectively, while in the USA these figures are 44 and 42% respectively [5, 6].

Several maternal and infant health problems have been related to excessive GWG and prepregnancy BMI [5–7], such as: (i) maternal comorbidities during pregnancy including gestational diabetes [8–11] and preeclampsia [9, 12]; (ii) delivery complications such as instrumental or cesarean delivery [3, 11–13]; (iii) being born large for gestational age [14]; and (iv) long-term effects in offspring such as adiposity [15] or lower cognitive skills [3, 16, 17].

Moreover, studies examining the compliance with the 1990 [6] or 2009 IOM GWG guidelines [5] and the WHO classification reported heterogeneous results in Europe and USA. The lack of compliance across countries could be explained by intercountry variability in several factors [18, 19] such as physical activity [20–22]. dietary patterns [21, 22], and psychological or social maternal characteristics [23–25].

For these reasons, this review aimed to estimate global and regional GWG (in kilograms) and the prevalence of GWG above, within and below the 2009 IOM guidelines. Second, we aimed to estimate prepregnancy BMI (in kg/m^2^) and the prevalence of BMI categories according to the WHO classification.

## Methods

This meta-analysis was registered in PROSPERO (registration number: CRD42018093562), and was reported following the Meta-analysis of Observational Studies in Epidemiology (MOOSE) statement [26]. The Cochrane Collaboration Handbook guidelines were used to guide this meta-analysis [27].

### Eligibility criteria, information sources, and search strategy

This systematic review and meta-analysis aimed to identify the studies reporting GWG published after the 2009 IOM guidelines. Due to the differences between 2009 IOM and 1990 guidelines, we decided to include only papers published following the 2009 IOM guidelines because differences in the classification of GWG categories could affect pooled GWG estimations. Studies were identified in the following databases: Medline (via PubMed), the Web of Science, the Cochrane Library and Embase (via Scopus) from 2009 (including studies published in 2009), when the IOM published the new cut-off points of GWG to 9 May 2018. The search strategy combined the following terms: (1) population (gestational, gestation, pregnancy, maternal) and (2) outcome (weight gain, weight change) (Table S1). The literature search was completed by screening the references included in the articles considered for inclusion in the systematic review.

### Study selection

The search was aimed to identify papers that reported GWG using the 2009 IOM cut-off points. Inclusion criteria were as follows: (i) participants: population-based pregnant studies of women with a singleton pregnancy; (ii) study design: observational studies; and (iii) outcomes: included studies had to report GWG mean and GWG classified with the 2009 IOM criteria [3]. Studies were excluded when they were written in languages other than English or Spanish or the target population was: (i) samples of a specific age-range; (ii) women with any specific prepregnancy weight status or BMI category (we excluded data of GWG and prepregnancy BMI categories of studies when they reported various categories combined in a data). (iii) women with any specific GWG category; (iv) women at any specific pregnancy trimester (v) pregnant women with specific diseases (diabetes mellitus, preeclampsia, cardiovascular health problems, anemia, gestational nausea and vomiting); (vi) pregnant women under pharmacological treatment during pregnancy or who underwent prepregnancy bariatric surgery; and (vii) only preterm deliveries. Additionally, studies were excluded when they used BMI classification criteria different from those of the WHO [2].

### Data synthesis

The following data were extracted from the original reports: (i) study data (author, the year of publication, country, regions, cohort year of birth, full term rate, sample size); (ii) characteristics of participants (mother’s age at delivery, mean GWG, the percentage of participants meeting the IOM guidelines, mean BMI and the prevalence of WHO BMI categories). When more than one study provided data from the same cohort and they did not overlapped, we included all available studies. However, when various studies provided data for the same cohort and they overlapped, we included only the one presenting the most detailed results or providing data with the largest sample size. However, data regarding sample characteristics could be extracted from all reports to obtain the most complete information.

**Fig. 1:**
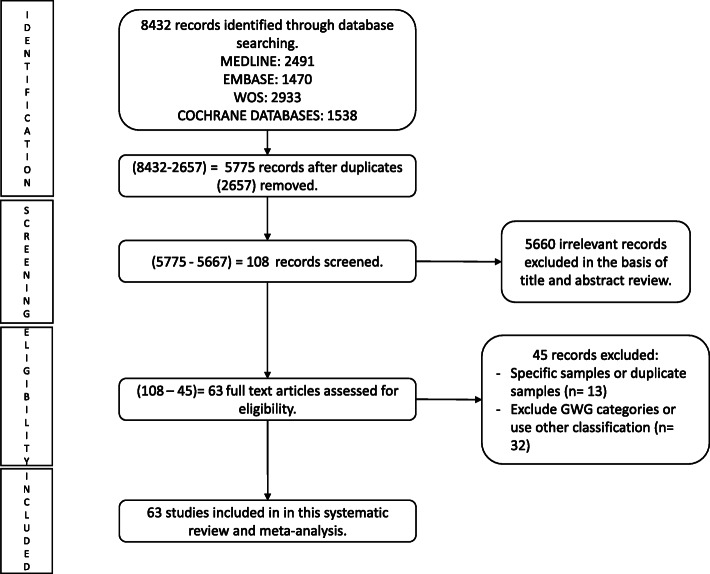
PRISMA flow chart

The search results were compiled in the Mendeley reference manager and the results of the systematic search are presented in Fig. 1. The characteristics of the included studies are presented in Table 1. DerSimonian and Laird random effects models [90] were used to compute global, and regional pooled estimates and their respective 95% confidence intervals (95% CIs) for: i) mean GWG, ii) prevalence of GWG categories according to the 2009 IOM guidelines (including below, within and above according to the 2009 IOM guidelines) [3] and iv) the prevalence of prepregnancy BMI according to the WHO BMI categories (including underweight, normal-weight and overweight BMI) [2].

**Table 1:** Characteristics of studies included

Reference	Country	Regions	Years of recruitment		Mean (SD) maternal age (years)	Full term rate (%)	n	Mean (SD) GWG (kg)	GWG according to 2009 IOM guidelines		Mean (SD) BMI (kg/m^**2**^)	Prepregnancy BMI according to WHO classification criteria			
									Below (%)	Above (%)		UW (%)	NW (%)	OW (%)	OB (%)
Asefa et al. 2016 [28]	Ethiopia	Africa	2014	2014	25.2 (5.01)	82.78	411	8.96 (3.27)	69.3	2.7	22.39 (3.84)	9.5	72	14.6	3.9
Guo et al. 2015 [29]	China	Asia	1992	1996	23.9 (2.1)	92.8	100,612	11.7 (5)	50.6	14.5	20.3 (2)	16.5	81.2	2.3^a^	
Abeysena et al. 2011 [30]	Sri Lanka	Asia	2001	2002	26.4 (5.5)	NA	481	10.6 (3.3)	58.5	8.7	NA	30	51.7	16^a^	
Munim et al. 2012 [31]	Pakistan	Asia	2003	2007	28.2 (4.8)	83.8	4735	8.5 (3.8)	52.4	13.5	NA	3.1	46.5	28.3	22.1
Shi et al. 2014 [32]	China	Asia	2006	2010	27.3 (4.2)	100	26,896	14.7 (4.6)	21.38	39.35	21 (2.9)	18.7	71.5	8.9	0.9
Radhakrishnan et al. 2014 [33]	India	Asia	2010	2011	27.3 (4.27)	88.03	1462	10.51 (4.46)	37.41	21.41	NA	7.18	47.06	34.54	11.22
Soltani et al. 2017 [34]	Indonesia	Asia	2010	2010	28.5 (5.6)	NA	607	10.2 (6)	56.14	11.72	21.3 (3.5)	20.1	65.3	13.5	1.1
Li et al. 2015 [35]	China	Asia	2011	2011	27.38 (4.46)	100	48,867	14.47 (4.98)	25	38.2	NA	13.15	76.45	9.2	1.2
Abbasalizad et al. 2016 [36]	Azerbaijan	Asia	2012	2013	27.15 (5.64)	NA	481	8.99 (5.87)	49.06	23.28	NA	2.5	41.16	39.92	16.42
Papazian et al. 2017 [37]	Lebanon	Asia	2012	2013	31.5 (4.4)	100	978	12.7 (6)	31.27	25.28	23 (3.8)	6.5	67.8	17.9	5.6
Kheirouri et al. 2017 [38]	Iran	Asia	2012	2014	NA	NA	717	10.5 (NA)	29.43	24.41	NA	3.9	44.35	35.15	16.6
Enomoto et al. 2016 [39]	Japan	Asia	2013	2013	31.8 (5.38)	89.27	97,157	9.97 (3.98)	63.82	7.07	19.64 (1.27)	18.24	71.15	7.72	28.87
Liu et al. 2015 [40]	China	Asia	2013	2014	NA	92.5	2973	13.72 (1.84)	13.6	53.8	22.29 (3.28)	8.5	72.4	19.1	0
Thapa et al. 2017 [41]	Nepal	Asia	2017	2017	26.15 (4.54)	100	227	10.21 (4.18)	42.7	15	NA	5.7	57.26	27.75	9.25
Mourtakos et al. 2017 [42]	Greece	Europe	1988	2000	27.84 (5.5)	100	5125	14.3 (7)	31.5	34.4	NA	3.8	78.9	14.8	2.5
Waters et al. 2012 [43]	UK	Europe	1990	2009	27.97 (5.81)	NA	439	12.21 (4.35)	19.8	48.3	28.12 (7.88)	44	22.3	33.7	0
Beyerlein et al. 2012 [44]	Germany	Europe	1996	2001	28.6 (4.8)	NA	6783	14.3 (6.1)	27.4	37	22.6 (3.8)	6.9	74.6	13.5	5
Ferrari et al. 2014 [5]	Germany	Europe	2000	2012	32.5 (5.4)	NA	11,678	13.3 (5.6)	27.4	36	24 (5)	5	65.8	19.1	10.2
Jacota et al. 2017 [45]	France	Europe	2003	2006	29.9 (4.5)	NA	1045	13.3 (4.9)	24.4	31.2	23.4 (4.6)	7.9	64.7	18	9.3
Henriksson et al. 2015 [46]	Sweden	Europe	2007	2010	31 (4)	100	312	15 (5)	18.3	45.2	23.1 (3.3)	1.9	75.3	17.6	5.1
Walsh et al. 2014 [47]	Ireland	Europe	2007	2011	31.81 (4.18)	100	621	12.19 (3.73)	5.9	43	27.09 (4.95)	0.3	41.1	38	20.6
Kinnunen et al. 2016 [48]	Norway	Europe	2008	2010	29.89 (4.53)	NA	623	13.64 (5.97)	25.2	42.22	24.46 (4.64)	NA	NA	NA	NA
Chmitorz et al. 2012 [49]	Germany	Europe	2009	2011	29 (4.55)	100	9824	15 (4.55)	17.68	47.1	23.5 (2.05)	4.7	69.64	18.19	7.49
Popa et al. 2014 [50]	Romania	Europe	2010	2010	27.53 (NA)	NA	400	14.24 (6.54)	22.7	35	22.31 (3.79)	11.8	66.6	17	4.6
Przybyłowicz et al. 2014 [51]	Polond	Europe	2010	2012	28.1 (NA)	100	510	15.4 (5.8)	16.6	47.4	22.4 (3.9)	10.6	70.9	18.5^a^	
Diemert et al. 2016 [52]	Germany	Europe	2011	2013	31 (3.5)	100	197	11.2 (3.9)	38	22	24.7 (4.6)	4	43	49	13
Heery et al. 2015 [53]	Ireland	Europe	2011	2011	31 (NA)	NA	799	15.54 (NA)	10	62.5	23.9 (5.82)	4.4	64.7	20.5	10.4
Vila-Candel et al. 2015 [54]	Spain	Europe	2011	2012	NA	100	140	14.63 (4.59)	16.4	45	21.92 (1.41)	7.14	67.86	21.43	3.57
Logan et al. 2017 [55]	Germany	Europe	2012	2013	32.5 (1.03)	100	949	14.65 (1.14)	16.1	48.5	NA	2.9	62.7	22.2	12.2
Özdek et al. 2015 [56]	Turkey	Europe	2012	2013	NA	100	208	11.81 (3.77)	20.7	38.9	28.12 (NA)	9.1	61.5	20.7	8.7
Cinelli et al. 2016 [57]	Italy	Europe	2013	2015	33 (1.33)	NA	435	13 (1)	28.5	33.1	21.9 (0.77)	6.4	71	15	7.6
Maier et al. 2016 [58]	Germany	Europe	2014	2014	NA	NA	591	14.03 (5.7)	19	44	NA	8	62.1	17.94	11.67
Ramón-Arbués et al. 2017 [59]	Spain	Europe	2014	2014	30.75 (4.29)	100	301	14.02 (2.33)	8.3	8.3	23.38 (3.92)	8.97	61.46	19.93	9.6
Tulmaç et al. 2018 [60]	Turkey	Europe	2015	2016	NA	100	163	11.86 (7.23)	16.6	60.7	25.47 (5.05)	3.68	50.3	26.99	19.02
Margerison et al. 2010 [61]	USA	North America	1972	2000	26.1 (5.5)	NA	4496	14.2 (6.9)	29.7	39.9	NA	7	69	16	8
Widen et al. 2015 [62]	USA	North America	1991	1993	26.4 (4.7)	100	156	15.6 (6.6)	16	56	24.5 (4.9)	3.9	60.3	23.7	12.2
Wander et al. 2015 [63]	USA	North America	1996	2008	32.6 (4.6)	100	3621	16.2 (5.7)	14	54	23.7 (5.1)	NA	NA	NA	NA
Larouche et al. 2010 [64]	Canada	North America	1998	2007	32.29 (4.76)	NA	960	16.56 (5.4)	9.3	64.6	24.6 (4.78)	2.9	59.4	25.2	12.5
Badon et al. 2014 [65]	USA/Canada	North America	2000	2006	30.4 (5.67)	100	5297	15.7 (6.39)	11.6	56.5	25 (5.37)	3.4	56.9	25	14.8
Deierlein et al. 2011 [66]	USA	North America	2001	2005	NA	100	361	16 (5.4)	13.07	56.82	24.2 (5.6)	5.93	65.25	16.38	12.43
Polinski et al. 2017 [67]	USA	North America	2001	2001	NA	85	6450	12.9 (0.1)	30.2	43.6	24.09 (0.05)	4.1	55.4	25.3	15.2
Ferraro et al. 2012 [68]	Canada	North America	2002	2009	30 (5.1)	NA	4321	16.13 (6.76)	13.1	57.63	25 (5.6)	3.9	56.2	23.7	16.2
Davis et al. 2014 [69]	USA	North America	2004	2008	NA	78.66	159,244	13.21 (NA)	24.96	40.78	25.51 (NA)	5.72	51.78	23.04	19.45
Fontaine et al. 2012 [70]	USA	North America	2004	2007	28.1 (5.3)	100	2760	13.2 (2.8)	18.91	48.26	NA	2.03	43.3	28.3	26.3
Park et al. 2011 [71]	USA	North America	2004	2007	NA	100	570,672	15.72 (6.94)	20.2	51.2	NA	4.9	53.5	23.8	17.8
Fuemmeler et al. 2016 [72]	USA	North America	2005	2011	27.96 (6.05)	89	734	14.26 (8.21)	22	56	NA	5	37	24	34
Gawade et al. 2011 [73]	USA	North America	2005	2008	27.38 (6.45)	100	2495	15.05 (7.34)	19.1	53.67	27.04 (6.4)	3.1	41.5	27.4	28
Kowal et al. 2011 [74]	Canada	North America	2005	2006	NA	NA	74,523	15.8 (2.71)	18.7	48.7	NA	6.1	59.4	21	13.5
Gallagher et al. 2014 [75]	USA	North America	2006	2011	23.9 (4.77)	NA	445	16.1 (6.2)	12	55	24.6 (5.3)	7.2	55	23	15
Simas et al. 2012 [76]	USA	North America	2006	2010	29 (6.1)	95.5	11,203	12.66 (3.38)	17.04	57.2	23.51 (1.7)	3.8	50.9	24.6	20.6
Pawlak et al. 2015 [77]	USA	North America	2007	2010	27.9 (6.1)	100	230,698	13.74 (6.53)	23.11	47.73	25.4 (5.7)	4.3	53.4	24.6	17.7
Ashley-Martin et al. 2016 [78]	Canada	North America	2008	2011	33 (5.17)	NA	1613	15.2 (8.05)	17.8	56.5	NA	2.7	61.6	21.6	14.1
Kominiarek et al. 2017 [79]	USA	North America	2009	2015	23.6 (5.2)	93.78	6351	13.15 (2.62)	24.3	49.9	26.77 (6.32)	4.22	41.82	27.95	26.01
Subhan et al. 2017 [80]	Canada	North America	2009	2012	NA	100	513	12.43 (1.99)	10.14	49.9	NA	3	65	19	12
Starling et al. 2015 [81]	USA	North America	2010	2013	NA	100	826	14.3 (6.6)	21	51	25.8 (NA)	3	52	25	20
Mamun et al. 2011 [82]	Australia	Oceania	1981	1983	25 (5.07)	96	6632	14.8 (5.2)	25	36	NA	9.9	74.3	11.7	4.2
Blumfield et al. 2015 [83]	Australia	Oceania	2006	2007	29 (5.6)	NA	134	13.3 (7)	26.87	40.3	NA	4.5	53	23.9	18.7
de Jersey et al. 2012 [84]	Australia	Oceania	2010	2011	29.9 (5.1)	100	569	13.4 (6.6)	26	38	24.3 (5.2)	5.8	60.1	21.8	11.42
Hartley et al. 2016 [85]	Australia	Oceania	2011	2011	32.3 (4.8)	NA	256	13.65 (5.77)	20.31	41.4	25.66 (5.97)	3.9	53.9	24.2	18
Castillo et al. 2016 [86]	Brazil	South America	2004	2004	26.1 (6.8)	NA	3757	12.4 (6.1)	30.4	33.2	24.2 (4.67)	4.8	60.9	23.3	10.9
Drehmer et al. 2010 [87]	Brazil	South America	2006	2007	25 (6.4)	NA	667	13.9 (6.5)	25.79	44.83	24.25 (4.7)	3.9	62.1	22.2	11.8
Paulino et al. 2016 [88]	Brazil	South America	2013	2014	26.69 (5.01)	100	290	11.38 (5.56)	32.07	31.38	NA	4.6	54.6	26.2	14.6
Garmendia et al. 2017 [89]	Chile	South America	2014	2015	25.8 (6.2)	100	1654	12.9 (6.2)	23.9	43.6	26.7 (5.3)	2.2	41.3	33.8	23.6

^a^ The study reported overweight and obesity categories in one category

*BMI* Body mass index, *SD* standard deviation, *UW* Underweight, *NW* Normal weight, *OW* Overweight, OB Obesity, *NA* Not Available

The heterogeneity of results across studies was evaluated by using the I^2^ statistic and could be considered: not important (0 to 40%), moderate (30 to 60%), substantial (50 to 90%) and considerable (75 to 100%); the corresponding *p*-values were also considered [91].

Furthermore, subgroup analyses by the method of collecting GWG or prepregnancy BMI data (self-reported by pregnant women, measured the GWG in the follow-up period or used data of medical records) were conducted for global and by regions estimations. In addition, random-effects meta-regressions estimations were conducted to evaluate the study and sample characteristics such as maternal age, year of the recruitment, full-term rate and country income (Tables S2, S3, S4, S5 and S6). In the analysis by country income we take into account the 2018 IMF list (Table S6), in which negative estimates mean that higher country’s income increases the dependent variable [92]. Statistical analyses were performed using StataSE software, version 15 (StataCorp).

### Assessment of risk bias

The Quality Assessment Tool for Observational Cohort and Cross-Sectional Studies was used to evaluate the risk of bias of the included studies [93]. In the Table S7 each yes added a point to the total score (+); and “no”, “cannot determine” (CD), “not applicable” (NA) or “not reported” (NR) kept the total score the same [93]. Each study could be scored as good (most methodological criteria met, low risk of bias), fair (some criteria met, low risk of bias), or poor (few criteria met, high risk of bias).

The literature search, data extraction and quality assessment were independently performed by two reviewers (JAM-H and IC-R), and inconsistencies were solved by consensus. A third researcher was consulted when consensus could not be reached (CA-B).

## Results

### Study selection

After screening 8432 articles, 63 studies were included in this systematic review and meta-analysis (Fig. 1). Included studies were published between 2010 and 2018, 21 were from North America [61–81], 20 were from Europe [5, 42–60], 13 were from Asia [29–41], four were from Oceania [82–85], four were from South America [86–89] and one was from Africa [28].

Studies were published from 2009 to 2017 [5, 28–89]. The population recruitment periods were 1 year in 12 studies [28, 34, 35, 39, 41, 50, 53, 58, 59, 67, 85, 86], 2 years in 15 studies [30, 33, 36, 37, 40, 54–56, 60, 74, 83, 84, 87–89] and more than 2 years in 36 studies [5, 29, 31, 32, 38, 42–49, 51, 52, 57, 61–66, 68–73, 75–82].

The number of participants was 1.416.915. The mean maternal age ranged from 23.6 to 33.0 years. Preterm delivery rates were reported by 40 studies ranging from 0.0 to 21.34% (Table 1) [28, 29, 31–35, 37, 39–42, 46, 47, 49, 51, 52, 54–56, 59, 60, 62, 63, 65–67, 69, 70, 72, 73, 76, 77, 79–82, 84, 88, 89]. Twenty studies did not report the mean BMI [30, 31, 33, 35, 36, 38, 41, 42, 55, 58, 61, 70–72, 74, 78, 80, 82, 83, 88] and only two studies did not report the prevalence of BMI categories [48, 63]. Furthermore, regarding the methods of collecting GWG: eighteen studies measured the GWG [30, 36, 43, 45–48, 52, 54, 57, 59, 60, 62, 63, 65, 78, 83, 84], thirty-eight used medical records [5, 28, 29, 31–35, 38–42, 44, 49–51, 53, 55, 56, 58, 61, 66, 68–72, 75, 77, 79, 81, 82, 85–89] and seven studies used women’s self-reported data [37, 64, 67, 73, 74, 80]. However, regarding the methods of collecting prepregnancy BMI data: only two studies measured it [52, 60], twelve used medical records [5, 28, 29, 39, 44, 49, 57, 68, 77, 79, 86, 89], two used both medical records and self-reported data [43, 76], and twenty four used self-reported prepregnancy BMI [29, 32, 34, 37, 45–48, 50, 51, 53, 54, 59, 62–67, 73, 75, 84, 85, 87].

### Synthesis of results

#### Gestational weight gain

Figure S1 display the estimations of GWG mean in the global population and by regions. The pooled GWG mean was 13.39 kg (95% CI: 11.97, 13.83) in the global population. Our findings showed that in global population 27.8% (95% CI: 26.5, 29.1) of women had GWG below guidelines and 39.4% (95% CI: 37.1, 41.7) GWG above guidelines.

Data by regions showed the highest pooled GWG mean in North America with 14.74 kg (95% CI: 13.97, 15.51) and the lowest in Asia with 11.36 kg (95% CI: 10.14, 12.58) (Figure S1). The highest pooled prevalence of GWG below the 2009 IOM guidelines was found in Asia with 39.4% (95% CI: 30.1, 49.6%) and the lowest in North America with 19.1% (95% CI: 16.1, 22.5%) (Figure S2). The pooled prevalence of GWG within the 2009 IOM guidelines was similar across regions (ranging from 33.3 to 37.8%), except in Africa and North America, with a prevalence of 29.0% (95% CI: 27.7, 30.3%) and 28.0% (95% CI: 23.9, 32.5%), respectively (Figure S3). Finally, data for pooled prevalence of GWG above the 2009 IOM guidelines showed the highest prevalence in North America and the lowest in Asia, 50.6% (95% CI: 46.2, 55.0%) and 20.2% (95% CI: 12.9, 30.2%), respectively (Figure S4).

#### Prepregnancy BMI

Figure S5 displays the estimations about the mean prepregnancy BMI in the global population and by regions. The pooled mean prepregnancy BMI was 23.08 kg/m^2^ (95%CI: 22.87, 23.30) in the global population. The highest mean prepregnancy BMI was found in South America with 25.05 kg (95% CI: 23.39, 26.72) and the lowest was found in Asia with 11.36 kg (95% CI: 10.14, 12.58). Furthermore, our findings showed a low prevalence of prepregnancy BMI classified as underweight with 5.5% (95% CI 5.2, 5.9). However, the prevalence of women with a prepregnancy BMI classified as overweight and obese was high with 23.0% (95% CI: 22.3, 23.7) and 16.3% (95% CI: 15.4, 17.3), respectively.

Data by region are shown in Figures S5, S6, S7, S8 and S9. The pooled prevalence of prepregnancy BMI categories showed that studies conducted in Asia reported the lowest pooled mean prepregnancy BMI with 21.24 kg/m^2^ (95% CI 20.76, 21.71), the highest prevalence of underweight with 11.1% (95% CI: 9.6, 12.7) and the lowest prevalence of obesity with 5.4% (95% CI: 2.4, 11.6). Finally, the highest prevalence of obesity was in North America with 17.6% (95% CI: 16.5, 18.7).

#### Subgroup analyses and meta-regressions

There were no differences among subgroups in the global population and across regions (*p* > 0.05) when subgroup analysis was based on the methods of collecting GWG and prepregnancy BMI (studies that measured them or those that used medical records).

The random-effects meta-regression model showed that GWG decreases in the global population, Europe and North America as maternal age increases. Additionally, mean GWG and the prevalence of GWG above guidelines increased when the year of recruitment was later in the global population, Europe and North America. Finally, GWG increased when there were higher full-term rates (*p* < 0.05) (Table S2).

The random-effects meta-regression model showed that prepregnancy BMI decreased as maternal age increased in Europe, North America and the global population. Furthermore, the mean prepregnancy BMI decreased in the global population and in Europe with a later year of recruitment. In contrast, prepregnancy BMI increased in North America with a later year of the recruitment (*p* < 0.05) (Table S3).

Tables S4 and S5 display the results of the meta-regression by the year of recruitment and prevalence of GWG and prepregnancy BMI categories. We found that the prevalence of GWG above guidelines increase in Europe, North America and global population with a more recent the year of recruitment, while the prevalence of GWG below guidelines decreased in Europe. Additionally, we found that the prevalence of underweight and normal weight decreased in all regions, while overweight and obesity increased with a later year of recruitment.

Finally, Table S6 shows that the mean GWG and prepregnancy BMI were higher in wealthy countries, as well as the prevalence of GWG above guidelines, overweight and obesity. Conversely, when countries’ income was lower the prevalence of GWG within and below guidelines, underweight and normal weight prevalence were higher.

#### Risk of bias of included studies

The methodological quality was good in 95% of the studies and fair in the remaining 5%. Assessors were not blinded to the exposure status of participants in any of the included studies. Furthermore, only 35 studies reported a participation rate of eligible women over 50% and 17 studies reported loss of follow-up rate less than 20% after baseline measures (Table S7).

## Discussion

### Main findings

Our findings display a global high prevalence of GWG above and below the 2009 IOM guidelines, 27.8 and 39.4%, respectively. Furthermore, the mean GWG and prevalence of GWG above guidelines have increased. Finally, there was a global high prevalence of overweight and obesity, 23.0 and 16.3%, respectively.

### Comparison with existing literature

Our pooled estimates of the mean GWG and prepregnancy BMI, as well as the prevalence of GWG above, within and below guidelines, are similar to those previously reported [5, 6, 94]. Moreover, the findings of a previous study [95] were in line with our data because it reported higher rates of GWG above guidelines and obesity in the USA and Europe than in Asia. Finally, previous evidence are in line with our findings because it suggests that the mean and prevalence of GWG above guidelines have increased [5, 6].

The high prevalence of GWG above guidelines may be a consequence of several factors: (i) lifestyle changes, such as lower level of physical activity during pregnancy or inadequate diet [96]; (ii) psychological and social maternal influences, such as low knowledge about the importance of gaining adequate GWG, emotional instability or locus of control [97]; and (iii) the global nutritional transition in recent decades, which has accelerated the consumption of processed food and prepared meals, which could produce higher rates of GWG above guidelines [98].

The differences in GWG and prepregnancy BMI across regions could be influenced by countries’ income because the rates of GWG above guidelines, overweight and obesity are higher in high- or middle-income countries [7]. However, differences between populations in the same countries could be due to by individual socioeconomic status because a larger fraction of the global overweight and obese populations become relatively poor in countries that are economically developed, while in lower−/middle- income countries, the rates of overweight and obesity are higher among wealthier individuals [99]. Moreover, the economic crisis could increase the probability to being obese because it could reduce diet quality among populations with fewer resources [100], and this fact could increase the GWG and prepregnancy BMI [3].

There were differences between the 2009 and 1990 IOM guidelines (Tables S8 and S9); therefore, we decided to use the 2009 IOM guidelines because rates of GWG categories could be affected if we included studies that reported results according both guidelines [1, 3]. As GWG and prepregnancy BMI have a multifactorial origin [3, 101], other factors were studied using subgroup analyses and meta-regressions. The relationship of maternal age and GWG agrees with previous studies supporting that older women have lower GWG in Europe and in the global population [19, 36]. The year of recruitment showed that GWG is rising in all regions, according to previous studies in all regions [5, 6]. Finally, higher full-term rates are positively related with GWG as GWG increases with the number of weeks of gestation, peaking at 37 weeks or more of gestation [3, 102]. Since, preterm birth rates could be higher in sub-Saharan African or South Asian countries than in European countries, this fact could explain why GWG was lower in these regions [103].

The variability in subgroup and meta-regression analyses could be explained by several factors influencing both GWG and prepregnancy BMI: (i) low knowledge about the importance of adequate GWG, including the women’s perception that they can not control their own weight by themselves. (low external locus of control) [25, 97, 104]; (ii) inadequate physical activity rates and dietary patterns [20–22]; (iii) low maternal education and professional class, maternal age, multiparity and minority ethnicity [105], and a lack of access to nutrition programs in low-income women [106]. Furthermore, national GWG guidelines and energy-intake guidelines could increase differences among regions because their guidelines varied around the world [107].

Our estimations of global GWG and prepregnancy BMI could help promote health interventions and programs among pregnant women and women of childbearing age because the American Congress of Obstetricians and Gynecologists (ACOG) emphasizes the importance of managing GWG and prepregnancy BMI through diet and physical activity counseling [108]. Thus, public health practitioners and policy-makers should explain to women the optimal GWG according to current guidelines to improve perinatal outcomes [3]. Routine medical visits during the prenatal period could be an opportunity for health-care providers to implement physical activity and diet counseling to ensure adequate GWG and prepregnancy BMI [101, 109, 110], although recent evidence calls into question the efficacy of dietary and lifestyle interventions to prevent the consequences of excessive GWG [111, 112] and the need to assess interventions in order to recommend the best option [113]. Regardless the inconclusive evidence about interventions based on diet and exercise, health-care providers should implement interventions based on diet and exercise due to the low adverse consequences of lifestyle interventions and their potential benefits.

### Strengths and limitations

Some limitations of this study that could compromise our results should be stated. First, there is a lack of data from some countries, because we did not retrieve studies that reported data on GWG and prepregnancy BMI; therefore, there is a lack of information from specific world areas. Second, differences in the sample characteristics and geographic locations of the included studies may increase heterogeneity, which could threaten the generalization of our results; for example a study published in 2018 suggested that IOM guidelines could be applicable only in the USA, western Europe and eastern Asia [95]; however, we used the same classification criteria for all regions of the world, in the way that was reported by the different studies. Third, we included studies with different rates of preterm birth, although when they reported in the methodology or limitation section that these rates were higher than those of the reference population or they only included preterm births, we excluded them to limit bias. Fourth, we could not evaluate publication bias due to the design of our study. Fifth, all included studies had observational designs; therefore, the drawing of causal inferences was not possible. Sixth, we included studies with self-reported weights, which produce an underreported prepregnancy and delivery weight or an overestimated GWG, although the magnitude of error could be small and it is a practical measurement approach [114]. Seventh, only studies written in English or Spanish were included and grey literature was not reviewed. Eight, the IOM classification criteria were not be suitable for the Asians population because there is a different cut-off for BMI categories. Ninth, we could not perform subgroup analyses by ethnic characteristics as data were heterogeneously reported by the studies. Finally, most of the literature was from Europe and America and the data from other regions were fewer, thus the small number of studies from Africa, South America and Oceania could limit the validity of our results and findings should be taken with caution.

To improve the strength of this article we decided to include studies published since 2009 (including 2009), because the studies published before this year cannot include the new IOM cut-off points published in 2009. However, we included studies with cohorts of women before 2009 that were classified with 2009 IOM cut-off points. We performed this because we aimed to improve comparability and generalization of our results. Furthermore, we excluded studies with specific samples of GWG or pregestational BMI because they could be overrepresented, and the ability to produce generalizable results could be limited.

## Conclusions

In summary, our study showed a global high prevalence of GWG above 2009 IOM guidelines and overweight/obesity, as well as an increase in mean GWG and the prevalence of GWG above guidelines. Thus, health practitioners and policy-makers should encourage a healthy GWG and prepregnancy BMI to improve perinatal outcomes, through novel lifestyle interventions in each clinical context. Notwithstanding, our data highlight the need for additional population-based studies, especially using the 2009 IOM guidelines.

## Supplementary information

**Additional file 1: Figure S1.** Mean gestational weight gain in kilograms. **Figure S2.** Prevalence of gestational weight gain below the 2009 IOM guidelines. **Figure S3.** Prevalence of gestational weight gain within guidelines according to the 2009 IOM guidelines. **Figure S4.** Prevalence of excessive gestational weight gain according to the 2009 IOM guidelines. **Figure S5.** Mean prepregnancy BMI in kilograms per meter squared. **Figure S6.** Prevalence of underweight prepregnancy BMI. **Figure S7.** Prevalence of normal-weight prepregnancy BMI. **Figure S8.** Prevalence of overweight prepregnancy BMI. **Figure S9.** Prevalence of obesity prepregnancy BMI. **Table S1.** Search strategy for MEDLINE. **Table S2.** Meta-regression with mean GWG. **Table S3.** Meta-regression with prepregnancy BMI mean. **Table S4.** Meta-regression with the prevalence of GWG categories by year of recruitment. **Table S5.** Meta-regression with prevalence the prepregnancy BMI categories by year of recruitment. **Table S6.** Meta-regression by economic position according to IMF staff estimates of each country. **Table S7.** Quality assessment with The Quality Assessment Tool for Observational Cohort and Cross-Sectional Studies. **Table S8.** The 1990 IOM guidelines for total weight gain during pregnancy. **Table S9.** The 2009 IOM guidelines for total weight gain during pregnancy.

## Data Availability

All data generated or analyzed during this study are included in this published article and its additional file.

## Abbreviations

BMI: Body mass index
CI: Confidence intervals
GWG: Gestational weight gain
IOM: Institute of Medicine
WHO: World Health Organization
MOOSE: Meta-analysis of Observational Studies in Epidemiology
ACOG: The American Congress of Obstetricians and Gynecologists
