# Supplementary material for: Monitoring gestational weight gain and prepregnancy BMI using the 2009 IOM guidelines in the global population: a systematic review and meta-analysis
Source: BMC Pregnancy Childbirth. 2020 Oct 27;20:649. doi: 10.1186/s12884-020-03335-7 (PMC7590483; doi:10.1186/s12884-020-03335-7)
Supplement: Supplementary file 1 — Additional file 1: Figure S1. Mean gestational weight gain in kilograms. Figure S2. Prevalence of gestational weight gain below the 2009 IOM guidelines. Figure S3. Prevalence of gestational weight gain within guidelines according to the 2009 IOM guidelines. Figure S4. Prevalence of excessive gestational weight gain according to the 2009 IOM guidelines. Figure S5. Mean prepregnancy BMI in kilograms per meter squared. Figure S6. Prevalence of underweight prepregnancy BMI. Figure S7. Prevalence of normal-weight prepregnancy BMI. Figure S8. Prevalence of overweight prepregnancy BMI. Figure S9. Prevalence of obesity prepregnancy BMI. Table S1. Search strategy for MEDLINE. Table S2. Meta-regression with mean GWG. Table S3. Meta-regression with prepregnancy BMI mean. Table S4. Meta-regression with the prevalence of GWG categories by year of recruitment. Table S5. Meta-regression with prevalence the prepregnancy BMI categories by year of recruitment. Table S6. Meta-regression by economic position according to IMF staff estimates of each country. Table S7. Quality assessment with The Quality Assessment Tool for Observational Cohort and Cross-Sectional Studies. Table S8. The 1990 IOM guidelines for total weight gain during pregnancy. Table S9. The 2009 IOM guidelines for total weight gain during pregnancy. [file 12884_2020_3335_MOESM1_ESM.docx]

# Supplementary material

**Figure S1. Mean gestational weight gain in kilograms.**

|  | Pooled estimate (95% CI) | | | | |  |
| --- | --- | --- | --- | --- | --- | --- |
|  | Studies | Total sample | Mean | Low limit | Upper limit |  |
| Africa | 1 | 411 | 8.96 | 8.64 | 9.28 |  |
| Asia | 12 | 274,717 | 11.36 | 10.14 | 12.58 |  |
| Europe | 19 | 39,815 | 13.60 | 13.17 | 14.04 |  |
| North America | 20 | 927,809 | 14.74 | 13.97 | 15.51 |  |
| Oceania | 4 | 7,519 | 13.85 | 12.90 | 14.79 |  |
| South America | 4 | 6,334 | 12.67 | 11.96 | 13.38 |  |
| Global population | 60 | 1,256,605 | 13.39 | 12.97 | 13.83 |  |

Confidence interval (CI),

**Figure S2. Prevalence of gestational weight gain below the 2009 IOM guidelines.**

|  |  | Pooled estimate (95% CI) | | | | |  |  |
| --- | --- | --- | --- | --- | --- | --- | --- | --- |
|  |  | Studies | Total sample | Prevalence | Low limit | Upper limit |  |  |
| Africa |  | 1 | 411 | 64.7 | 73.6 | 69.3 |  |  |
| Asia |  | 13 | 275,484 | 30.1 | 49.6 | 39.4 |  |  |
| Europe |  | 20 | 40,614 | 16.9 | 22.7 | 19.6 |  |  |
| North America |  | 21 | 1,086,553 | 16.1 | 22.5 | 19.1 |  |  |
| Oceania |  | 4 | 7,519 | 23.5 | 26.7 | 25.1 |  |  |
| South America |  | 4 | 6,334 | 23.9 | 31.9 | 27.7 |  |  |
| Global population |  | 63 | 1,416,915 | 0.265 | 0.291 | 0.278 |  |  |

Confidence interval (CI)

**Figure S3. Prevalence of gestational weight gain within guidelines according to the 2009 IOM guidelines.**

|  |  | Pooled estimate (95% CI) | | | | | |  |
| --- | --- | --- | --- | --- | --- | --- | --- | --- |
|  |  | Studies | Total sample | | Prevalence | Low limit | Upper limit |  |
| Africa |  | 1 |  | 411 | 28.0 | 23.9 | 32.5 |  |
| Asia |  | 13 |  | 275,484 | 35.9 | 33.5 | 38.4 |  |
| Europe |  | 20 |  | 40,614 | 37.3 | 35.5 | 39.2 |  |
| North America |  | 21 |  | 1,086,553 | 29.0 | 27.7 | 30.3 |  |
| Oceania |  | 4 |  | 7,519 | 37.8 | 35.6 | 40.1 |  |
| South America |  | 4 |  | 6,334 | 33.6 | 30.4 | 37.0 |  |
| Global population |  | 63 |  | 1,416,915 | 33.3 | 32.5 | 34.2 |  |

Confidence interval (CI),

**Figure S4. Prevalence of excessive gestational weight gain according to the 2009 IOM guidelines.**

|  |  | Pooled estimate (95% CI) | | | | | |  |
| --- | --- | --- | --- | --- | --- | --- | --- | --- |
|  |  | Studies | Total sample | | Prevalence | Low limit | Upper limit |  |
| Africa |  | 1 | 411 |  | NA | NA | NA |  |
| Asia |  | 13 | 275,484 |  | 16.8 | 7.8 | 32.6 |  |
| Europe |  | 20 | 40,614 |  | 40.2 | 36.2 | 44.3 |  |
| North America |  | 21 | 1,086,553 |  | 50.3 | 45.4 | 55.1 |  |
| Oceania |  | 4 | 7,519 |  | 36.2 | 33.4 | 39.0 |  |
| South America |  | 4 | 6,334 |  | 38.7 | 28.1 | 50.6 |  |
| Global population |  | 63 | 1,416,915 |  | **39.7** | **37.7** | **41.7** |  |

Confidence interval (CI), not available (NA).

**Figure S5. Mean prepregnancy BMI in kilograms per meter squared.**

|  |  | Pooled estimate (95% CI) | | | | | |  |
| --- | --- | --- | --- | --- | --- | --- | --- | --- |
|  |  | Studies | Total sample | | Mean | Low limit | Upper limit |  |
| Africa |  | 1 | 411 |  | 22.39 | 22.02 | 22.76 |  |
| Asia |  | 6 | 272,871 |  | 21.24 | 20.76 | 21.71 |  |
| Europe |  | 15 | 33,308 |  | 23.84 | 23.33 | 24.34 |  |
| North America |  | 13 | 1,084,118 |  | 24.77 | 24.32 | 25.22 |  |
| Oceania |  | 2 | 825 |  | 24.95 | 23.62 | 26.28 |  |
| South America |  | 3 | 6,078 |  | 25.05 | 23.39 | 26.72 |  |
| Global population |  | 40 | 1,397,611 |  | 23.08 | 22.87 | 23.30 |  |

Confidence interval (CI).

**Figure S6. Prevalence of underweight prepregnancy BMI.**

|  |  | Pooled estimate (95% CI) | | | | | |  |
| --- | --- | --- | --- | --- | --- | --- | --- | --- |
|  |  | Studies | Total sample | | Prevalence | Low limit | Upper limit |  |
| Africa |  | 1 |  | 411 | 9.5 | 7.0 | 12.7 |  |
| Asia |  | 13 |  | 286,193 | 11.1 | 9.6 | 12.7 |  |
| Europe |  | 18 |  | 40,081 | 5.8 | 4.9 | 6.8 |  |
| North America |  | 20 |  | 1,084,118 | 4.4 | 4.0 | 4.7 |  |
| Oceania |  | 4 |  | 7,591 | 6.0 | 3.7 | 9.8 |  |
| South America |  | 4 |  | 6,368 | 3.5 | 2.4 | 5.2 |  |
| Global population |  | 60 |  | 1,424,762 | 5.5 | 5.2 | 5.9 |  |

Confidence interval (CI)

**Figure S7. Prevalence of normal-weight prepregnancy BMI.**

|  |  | Pooled estimate (95% CI) | | | | | |  |
| --- | --- | --- | --- | --- | --- | --- | --- | --- |
|  |  | Studies | Total sample | | Prevalence | Low limit | Upper limit |  |
| Africa |  | 1 |  | 411 | 72.4 | 67.5 | 76.1 |  |
| Asia |  | 12 |  | 284,731 | 64.1 | 59.0 | 69.0 |  |
| Europe |  | 17 |  | 40,0081 | 63.4 | 59.6 | 67.1 |  |
| North America |  | 20 |  | 1,084,118 | 54.1 | 52.7 | 55.6 |  |
| Oceania |  | 4 |  | 7591 | 61.3 | 48.5 | 72.6 |  |
| South America |  | 4 |  | 6368 | 52.2 | 40.4 | 63.7 |  |
| Global population |  | 58 |  | 1,424,762 | 56.7 | 55.5 | 58.0 |  |

Confidence interval (CI).

**Figure S8. Prevalence of overweight prepregnancy BMI.**

|  |  | Pooled estimate (95% CI) | | | | | |  |
| --- | --- | --- | --- | --- | --- | --- | --- | --- |
|  |  | Studies | Total sample | | Prevalence | Low limit | Upper limit |  |
| Africa |  | 1 |  | 411 | 14.6 | 11.5 | 18.4 |  |
| Asia |  | 10 |  | 182,538 | 19.7 | 14.3 | 26.5 |  |
| Europe |  | 19 |  | 40,520 | 19.5 | 17.5 | 21.7 |  |
| North America |  | 20 |  | 1,084,118 | 23.5 | 22.8 | 24.3 |  |
| Oceania |  | 4 |  | 7,591 | 19.6 | 12.4 | 29.5 |  |
| South America |  | 4 |  | 6,368 | 26.8 | 21.5 | 32.9 |  |
| Global population |  | 58 |  | 1,321,135 | 23.0 | 22.3 | 23.7 |  |

Confidence interval (CI);

**Figure S9. Prevalence of obesity prepregnancy BMI.**

|  |  | Pooled estimate (95% CI) | | | | | |  |
| --- | --- | --- | --- | --- | --- | --- | --- | --- |
|  |  | Studies | Total sample | | Prevalence | Low limit | Upper limit |  |
| Africa |  | 1 |  | 411 | 3.9 | 2.4 | 6.3 |  |
| Asia |  | 10 |  | 182,127 | 5.4 | 2.4 | 11.6 |  |
| Europe |  | 18 |  | 40,010 | 9.1 | 6.9 | 11.8 |  |
| North America |  | 20 |  | 1,084,118 | 17.6 | 16.5 | 18.7 |  |
| Oceania |  | 4 |  | 7,591 | 11.3 | 4.9 | 24.3 |  |
| South America |  | 4 |  | 6,368 | 16.4 | 10.1 | 25.6 |  |
| Global population |  | 57 |  | 1,320,625 | 16.3 | 15.4 | 17.3 |  |

Confidence interval (CI)

**Table S1. Search strategy for MEDLINE.**

| gestation*  OR  pregnancy  OR  maternal | AND | “weight gain”  OR  “weight change” | NOT | diabetes  OR  preeclampsia  OR  preterm  OR  hyperemesis  OR  stillbirth  OR  bariatric surgery  OR  postpartum  OR  offspring | NOT | meta-analysis  OR  systematic review |
| --- | --- | --- | --- | --- | --- | --- |

| **Table S2. Meta-regression with GWG mean.** | | | | | |
| --- | --- | --- | --- | --- | --- |
| Meta-regression by | Continent (number of studies) | Slope | LL | UL | p |
| Maternal age | Europe (16) | -0.20 | -0.22 | -0.18 | 0.00 |
|  | North America (15) | 0.16 | 0.15 | 0.17 | 0.00 |
|  | Global population (51) | -0.18 | -0.19 | -0.18 | 0.00 |
| Year of recruitment | Europe (20) | 0.02 | 0.01 | 0.02 | 0.00 |
|  | North America (21) | 0.37 | 0.36 | 0.37 | 0.00 |
|  | Global population (63) | 0.04 | 0.04 | 0.04 | 0.00 |
| Term rates | Europe (13) | NA | NA | NA | NA |
|  | North America (15) | 0.14 | 0.14 | 0.14 | 0.00 |
|  | Global population (29) | 0.11 | 0.10 | 0.11 | 0.00 |

Upper limit (UL), lower limit (LL).

| **Table S3. Meta-regression with prepregnancy BMI mean.** | | | | | |
| --- | --- | --- | --- | --- | --- |
| **Meta-regression by** | **Continent** | **Slope** | **LL** | **UL** | **p** |
| Maternal age | Europe (14) | -1.05 | -1.07 | -1.04 | 0.00 |
|  | North America (10) | -0.56 | -0.58 | -0.55 | 0.00 |
|  | Global population (35) | -0.14 | -0.14 | -0.13 | 0.00 |
| Year of recruitment | Europe (17) | -0.04 | -0.04 | -0.03 | 0.00 |
|  | North America (15) | 0.13 | 0.13 | 0.14 | 0.00 |
|  | Global population (44) | -0.20 | -0.20 | 0.20 | 0.00 |

Upper limit (UL), lower limit (LL).

| **Table S4. Meta-regression with the prevalence of GWG categories by year of recruitment.** | | | | | |
| --- | --- | --- | --- | --- | --- |
| Meta-regression by | Continent (number of studies) | Slope | LL | UL | p |
| GWG above guidelines | Europe (20) | 0.02 | 0.02 | 0.03 | 0.00 |
|  | North America (21) | 0.00 | 0.00 | 0.00 | 0.00 |
|  | Global population (63) | 0.01 | 0.01 | 0.01 | 0.00 |
| GWG within guidelines | Europe (20) | 0.01 | 0.00 | 0.01 | 0.00 |
|  | North America (21) | -0.00 | -0.01 | -0.00 | 0.00 |
|  | Global population (63) | -0.01 | -0.01 | -0.01 | 0.00 |
| GWG below guidelines | Europe (20) | -0.03 | -0.04 | -0.03 | 0.00 |
|  | North America (21) | -0.00 | -0.00 | 0.00 | 0.29 |
|  | Global population (63) | 0.00 | 0.00 | 0.00 | 0.00 |

Upper limit (UL), lower limit (LL).

| **Table S5. Meta-regression with the prevalence of prepregnancy BMI categories by year of recruitment.** | | | | | |
| --- | --- | --- | --- | --- | --- |
| Meta-regression by | Continent (number of studies) | Slope | LL | UL | p |
| Underweight | Europe (19) | -0.02 | -0.03 | -0.02 | 0.00 |
|  | North America (20) | -0.02 | -0.02 | -0.01 | 0.00 |
|  | Global population (61) | - 0.00 | -0.00 | -0.00 | 0.00 |
| Normal weight | Europe (19) | -0.02 | -0.02 | -0.02 | 0.00 |
|  | North America (20) | -0.02 | -0.01 | -0.02 | -0.00 |
|  | Global population (61) | -0.02 | -0.02 | -0.02 | 0.00 |
| Overweight | Europe (18) | 0.02 | 0.01 | 0.02 | 0.00 |
|  | North America (20) | 0.01 | 0.01 | 0.02 | 0.00 |
|  | Global population (58) | -0.03 | -0.03 | -0.02 | 0.00 |
| Obesity | Europe (18) | 0.03 | 0.03 | 0.04 | 0.00 |
|  | North America (20) | 0.02 | 0.02 | 0.02 | 0.00 |
|  | Global population (58) | 0.05 | 0.05 | 0.05 | 0.00 |

Upper limit (UL), lower limit (LL).

| **Table S6. Meta-regression by economic position according to IMF staff estimates of each country.** | | | | | |
| --- | --- | --- | --- | --- | --- |
| Meta-regression by | Number of studies | Slope | LL | UL | p |
| GWG mean | 60 | -0.00 | -0.00 | -0.00 | 0.00 |
| GWG above guidelines | 63 | -0.02 | -0.02 | -0.02 | 0.00 |
| GWG within guidelines | 63 | 0.00 | 0.00 | 0.00 | 0.00 |
| GWG below guidelines | 63 | 0.01 | 0.01 | 0.01 | 0.00 |
| Prepregnancy BMI mean | 40 | -0.08 | -0.08 | -0.08 | 0.00 |
| Underweight | 61 | 0.02 | 0.02 | 0.02 | 0.00 |
| Normal weight | 61 | 0.01 | 0.01 | 0.02 | 0.00 |
| Overweight | 59 | -0.01 | -0.01 | -0.01 | 0.00 |
| Obesity | 58 | -0.00 | -0.00 | -0.00 | 0.00 |

Upper limit (UL), lower limit (LL).

**Table S7. Quality assessment with The Quality Assessment Tool for Observational Cohort and Cross-Sectional Studies.**

|  | **Items*** | | | | | | | | | | | | | |  |  |
| --- | --- | --- | --- | --- | --- | --- | --- | --- | --- | --- | --- | --- | --- | --- | --- | --- |
| **First author/Item** | **1** | **2** | **3** | **4** | **5** | **6** | **7** | **8** | **9** | **10** | **11** | **12** | **13** | **14** | **Quality**  **score** | **Quality**  **score** |
| Asefa et al. 2016 | + | + | - | + | - | + | + | + | + | - | + | - | NA | + | 9 | Fair |
| Guo et al. 2015 | + | + | NR | + | - | + | + | + | + | + | + | + | + | + | 12 | Good |
| Abeysena et al. 2011 | + | + | - | + | - | + | + | + | + | + | + | + | + | + | 12 | Good |
| Munim et al. 2012 | + | + | NR | + | - | + | + | + | + | + | + | - | - | + | 10 | Good |
| Shi et al. 2014 | + | + | NR | + | - | + | + | + | + | + | + | - | NR | + | 10 | Good |
| Radhakrishnan et al. 2014 | + | + | NR | + | - | + | + | + | + | + | + | - | NA | + | 10 | Good |
| Soltani et al. 2017 | + | + | + | - | - | + | + | + | + | + | + | - | - | + | 10 | Good |
| Li et al. 2015 | + | + | + | + | - | + | + | + | + | + | + | - | NA | + | 11 | Good |
| Abbasalizad et al. 2016 | + | + | NR | - | - | + | + | + | + | + | + | - | NA | + | 9 | Fair |
| Papazian et al. 2017 | + | + | + | - | - | + | + | + | + | - | + | - | NA | + | 9 | Fair |
| Kheirouri et al. 2017 | + | + | NR | + | - | + | + | + | + | + | + | - | NA | + | 10 | Good |
| Enomoto et al. 2016 | + | + | + | + | - | + | + | + | + | + | + | - | NA | + | 11 | Good |
| Liu et al. 2015 | + | - | + | + | - | + | + | + | + | + | + | - | NA | + | 10 | Good |
| Thapa et al. 2017 | + | + | NR | + | - | + | + | + | + | + | + | - | NA | + | 10 | Good |
| Mourtakos et al. 2017 | + | + | - | + | - | + | + | + | + | + | + | - | NA | + | 10 | Good |
| Waters et al. 2012 | + | + | NR | + | - | + | + | + | + | + | + | - | NR | + | 10 | Good |
| Beyerlein et al. 2012 | + | + | + | + | - | + | + | + | + | + | + | - | - | + | 11 | Good |
| Ferrari et al. 2014 | + | + | NR | + | - | + | + | + | + | + | + | - | NR | + | 10 | Good |
| Jacota et al. 2017 | + | + | + | + | - | + | + | + | + | + | + | - | - | + | 11 | Good |
| Henriksson et al. 2015 | + | + | NR | + | - | + | + | + | + | + | + | - | NR | + | 10 | Good |
| Walsh et al. 2014 | + | + | + | + | - | + | + | + | + | + | + | - | + | + | 12 | Good |
| Kinnunen et al. 2016 | + | + | + | + | - | + | + | + | + | + | + | - | - | + | 11 | Good |
| Chmitorz et al. 2012 | + | + | + | + | - | + | + | + | + | + | + | - | + | + | 12 | Good |
| Popa et al. 2014 | + | + | NR | + | - | + | + | + | + | + | + | - | NA | + | 10 | Good |
| Przybyłowicz et al. 2014 (58) | + | + | + | + | - | + | + | + | + | + | + | - | NA | + | 11 | Good |
| Diemert et al. 2016 | + | + | NR | + | - | + | + | + | + | + | + | - | NR | + | 10 | Good |
| Heery et al. 2015 | + | + | + | + | - | + | + | + | + | + | + | - | + | + | 12 | Good |
| Vila-Candel et al. 2015 | + | + | NR | + | - | + | + | + | + | + | + | - | + | + | 11 | Good |
| Logan et al. 2017 | + | + | NR | + | - | + | + | + | + | + | + | - | - | + | 10 | Good |
| Özdek et al. 2015 | + | + | NR | + | - | + | + | + | + | + | + | - | NA | + | 10 | Good |
| Cinelli et al. 2016 | + | + | NR | + | - | + | + | + | + | + | + | - | - | + | 10 | Good |
| Maier et al. 2016 | + | + | NR | + | - | + | + | + | + | + | + | - | NR | + | 10 | Good |
| Ramón-Arbués et al. 2017 | + | + | - | + | - | + | + | + | + | + | + | - | + | + | 11 | Good |
| Tulmaç et al. 2018 | + | + | NR | + | - | + | + | + | + | + | + | + | NA | + | 11 | Good |
| Margerison et al. 2010 | + | + | NR | + | - | + | + | + | + | + | + | - | NR | + | 10 | Good |
| Widen et al. 2015 | + | + | NR | + | - | + | + | + | + | + | + | - | NR | + | 10 | Good |
|  | **Items*** | | | | | | | | | | | | | |  |  |
| **First author/Item** | **1** | **2** | **3** | **4** | **5** | **6** | **7** | **8** | **9** | **10** | **11** | **12** | **13** | **14** | **Quality**  **score** | **Quality**  **score** |
| Wander et al. 2015 | + | + | + | + | - | + | + | + | + | + | + | - | + | + | 12 | Good |
| Larouche et al. 2010 | + | + | + | + | - | + | + | + | + | + | + | - | NR | + | 11 | Good |
| Badon et al. 2014 | + | + | - | + | - | + | + | + | + | + | + | - | - | + | 10 | Good |
| Deierlein et al. 2011 | + | + | + | + | - | + | + | + | + | + | + | - | - | + | 11 | Good |
| Polinski et al. 2017 | + | + | - | + | + | + | + | + | + | + | + | + | - | + | 12 | Good |
| Ferraro et al. 2012 | + | + | + | + | - | + | + | + | + | + | + | - | - | + | 11 | Good |
| Davis et al. 2014 | + | + | + | + | - | + | + | + | + | + | + | - | + | + | 12 | Good |
| Fontaine et al. 2012 | + | + | + | + | - | + | + | + | + | + | + | - | + | + | 12 | Good |
| Park et al. 2011 | + | + | + | + | - | + | + | + | + | + | + | - | - | + | 11 | Good |
| Fuemmeler et al. 2016 | + | + | - | + | - | + | + | + | + | + | + | - | - | + | 10 | Good |
| Gawade et al. 2011 | + | + | + | + | - | + | + | + | + | + | + | - | - | + | 11 | Good |
| Kowal et al. 2011 | + | + | + | + | - | + | + | + | + | + | + | - | - | + | 11 | Good |
| Gallagher et al. 2014 | + | + | + | + | - | + | + | + | + | + | + | - | - | + | 11 | Good |
| Simas et al. 2012 | + | + | + | + | - | + | + | + | + | + | + | - | + | + | 12 | Good |
| Pawlak et al. 2015 | + | + | + | + | - | + | + | + | + | + | + | - | + | + | 12 | Good |
| Ashley-Martin et al. 2016 | + | + | + | + | - | + | + | + | + | + | + | - | + | + | 12 | Good |
| Kominiarek et al. 2017 | + | + | + | + | - | + | + | + | + | + | + | - | NR | + | 11 | Good |
| Subhan et al. 2017 | + | + | + | + | - | + | + | + | + | + | + | - | + | + | 12 | Good |
| Starling et al. 2015 | + | + | + | + | - | + | + | + | + | + | + | - | - | + | 11 | Good |
| Mamun et al. 2011 | + | + | + | + | - | + | + | + | + | + | + | - | + | + | 12 | Good |
| Blumfield et al. 2015 | + | + | + | + | - | + | + | + | + | + | + | - | - | + | 11 | Good |
| de Jersey et al. 2012 | + | + | + | + | - | + | + | + | + | + | + | - | + | - | 11 | Good |
| Hartley et al. 2016 | + | + | + | + | - | + | + | + | + | + | + | - | - | + | 11 | Good |
| Castillo et al. 2016 | + | + | + | + | - | + | + | + | + | + | + | - | + | + | 12 | Good |
| Drehmer et al. 2010 | + | + | + | + | - | + | + | + | + | + | + | - | + | + | 12 | Good |
| Paulino et al. 2016 | + | + | NR | + | - | + | + | + | + | + | + | - | NR | + | 10 | Good |
| Garmendia et al. 2017 | + | + | + | + | - | + | + | + | + | + | + | - | - | + | 11 | Good |

Yes (+), no (-), not applicable (NA) or not reported (NR); *Items description of The Quality Assessment Tool for Observational Cohort and Cross-Sectional Studies:

1. Was the research question or objective in this paper clearly stated?

2. Was the study population clearly specified and defined?

3. Was the participation rate of eligible persons at least 50%?

4. Were all the subjects selected or recruited from the same or similar populations (including the same time period)? Were inclusion and exclusion criteria for being in the study prespecified and applied uniformly to all participants?

5. Was a sample size justification, power description, or variance and effect estimates provided?

6. For the analyses in this paper, were the exposure(s) of interest measured prior to the outcome(s) being measured?

7. Was the timeframe sufficient so that one could reasonably expect to see an association between exposure and outcome if it existed?

8. For exposures that can vary in amount or level, did the study examine different levels of the exposure as related to the outcome (e,g,, categories of exposure, or exposure measured as continuous variable)?

9. Were the exposure measures (independent variables) clearly defined, valid, reliable, and implemented consistently across all study participants?

10. Was the exposure(s) assessed more than once over time?

11. Were the outcome measures (dependent variables) clearly defined, valid, reliable, and implemented consistently across all study participants?

12. Were the outcome assessors blinded to the exposure status of participants?

13. Was loss to follow-up after baseline 20% or less?

14. Were key potential confounding variables measured and adjusted statistically for their impact on the relationship between exposure(s) and outcome(s)?,

**Table S8. The 1990 IOM guidelines for total weight gain during pregnancy.**

| **Recommended total gestational weight gain** | |
| --- | --- |
| **BMI category** | **Kg** |
| <19.8 | 12.5-18 |
| 19.8-26 | 11.5-16 |
| 26.0-29.0 | 7-11.5 |
| >29 | ≥ 6.8 |

**Table S9. The 2009 IOM guidelines for total weight gain during pregnancy.**

| **Recommended total gestational weight gain** | |
| --- | --- |
| **BMI category** | **Kg** |
| <18.5 | 12.5-18 |
| 18.5-24.9 | 11.5-16 |
| 25.0-29.9 | 7-11.5 |
| 30 | 5-9 |
